# A rare case report of bilateral Purtscher-like retinopathy in juvenile dermatomyositis

**DOI:** 10.3205/oc000237

**Published:** 2024-05-07

**Authors:** Nidhi Paharia, Shruti Agrawal, Nikhil Agrawal, Jayesh Shah

**Affiliations:** 1 Department of Ophthalmology, All India Institute of Medical Sciences (AIIMS), Bhatinda, Punjab, India; 2 Department of Pathology, All India Institute of Medical Sciences (AIIMS), Rishikesh, Uttarakhand, India; 3 Department of Ophthalmology, All India Institute of Medical Sciences (AIIMS), Bathinda, Punjab, India; 4 Department of Pathology, Christian Medical College, Vellore, India

**Keywords:** Purtscher-like retinopathy, Purtscher flecken, pneumonitis, juvenile dermatomyositis

## Abstract

**Purpose:**

To report a rare case of bilateral Purtscher-like retinopathy (PLR) in a young adult diagnosed with dermatomyositis

**Method:**

A case report with multi-modal imaging

**Result:**

A 17-year-old male presented with subacute marked diminution of vision along with arthralgia, weakness of all four limbs and development of multiple rashes around body. Fundus examination revealed bilateral multiple Purtscher flecken, pseudo-cherry red spot, and intra-retinal haemorrhages with cotton wool spots. Systemic and laboratory examinations, magnetic resonance imaging (MRI) and biopsy of tissue confirmed the diagnosis of juvenile dermatomyositis with PLR.

**Conclusion:**

Dermatomyositis, being a rare cause of PLR, should essentially be considered as one of the differentials as timely intervention can alter the course of disease and prove life-saving for the patient.

## Introduction

Juvenile dermatomyositis (JDM) is a rare multi-system autoimmune disorder with a global incidence ranging from 1.9 to 4.1 cases per million children per year [1], [2]. It is characterised by inflammation of the skin and skeletal muscle with concurrent involvement of the heart, lungs and gastrointestinal tract. The aetiology of JDM is largely unknown, however in retrospective studies, a history of infection prior to the onset of disease has been elicited in a large proportion of patients. This has led to the hypothesis that in patients with genetic susceptibility, an infectious etiology may be the triggering factor in the onset of disease [2]. Furthermore, association with Purtscher-like retinopathy (PLR) is a rare occurrence in JDM. Herein, we report an unusual case of a young male diagnosed with dermatomyositis presenting primarily with PLR, with a preceding history of a respiratory tract infection.

## Case description

A 17-year-old male presented to us with subacute, painless, progressive, marked diminution of vision in both eyes for the last month which was preceded by an episode of upper respiratory tract infection. This was followed by arthralgia, weakness of upper and lower limbs associated with difficulty in carrying out daily activities like combing hair, squatting and an inability to stand up from a sitting position. Subsequently, he developed hoarseness of voice along with difficulty in deglutition and itchy rashes around eyes, neck, chest, back, arms and legs over the last 2 weeks. 

The patient revealed a past history of fungal retinitis for which he was started on oral voriconazole. However, the lack of any improvement in his condition eventually led to his referral to a higher centre for further management. The systemic history of the patient was unremarkable.

**Figure 1:**
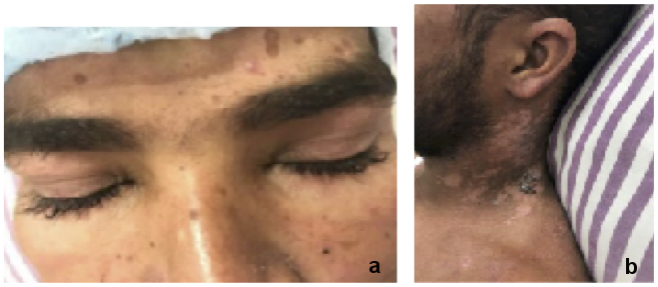
(a) Violaceous eruptions present around eyelids – heliotrope rash; (b) cutaneous lesions in healing stage

On general examination, there was presence of erythematous macular papular lesions over the neck (Figure 1b (Fig. 1)), lower back, extensor surface of arm, shin of tibia and small joints of hands. Neurological examination showed significant wasting and weakness of the proximal muscles of both upper (power=3/5) and lower limbs (power=2/5). Deep tendon reflexes were diminished but sensory examination was within normal limits.

On ophthalmic examination, the patient had heliotrope rashes in the form of violaceous discolouration along with mild swelling over the upper lids of both eyes (Figure 1a (Fig. 1)). The best corrected visual acuity was finger counting close to face (FCCF) in both eyes. Pupillary reactions were sluggish and with no relative apparent pupillary defect (RAPD). Extra-ocular movements were full and there was no subjective complaint of pain on movement. 

**Figure 2:**
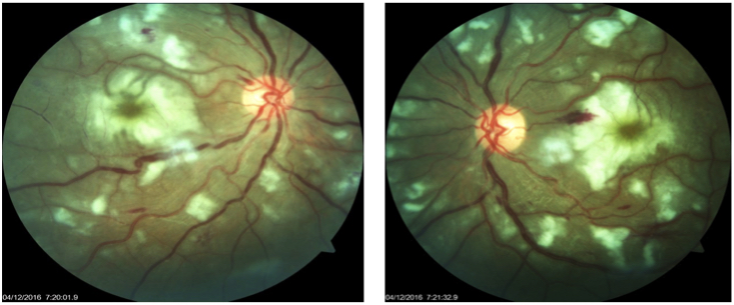
Fundus photograph showing multiple Purtscher flecken, scattered foci of cotton wool spots, intra retinal haemorrhages, pseudo-cherry red spot with macular oedema and engorged retinal vessels

Slit-lamp bi-microscopy examination showed unremarkable anterior segment findings and intra-ocular pressure. On fundus examination, there was mild temporal pallor of the disc in both eyes. The right eye showed splinter haemorrhages at the supero-temporal margin of the disc. Multiple Purtscher flecken (Figure 2 (Fig. 2)) and polygonal areas of retinal whitening were present around peripapillary area in zone 1 (4 DD radius area around the optic nerve head). An area of whitening surrounding the macular oedema commonly referred to as “pseudo-cherry red spot” was identified at the macula. Multiple cotton wool spots and intra-retinal haemorrhages were observed in both eyes in zone 1. Vessels were dilated and tortuous but there were no visible emboli in retinal vessels. A diagnosis of bilateral PLR was made and the patient was referred for neurological and dermatological opinion. Ocular coherence tomography and fundus fluorescein angiography could not be done due to progressively deteriorating general condition of the patient accompanied by the unfavourable blood parameters.

Laboratory examination showed an elevated total leukocyte count (TLC) of 18.2x10^9^/L, differential count of neutrophils 90%, lymphocytes 7%, monocytes 3% and a reduced platelet count of 64x10^9^/L. ESR and C-reactive protein were raised with values of 28 mm/hour and 8.9 mg/dl, respectively. The liver enzymes were deranged with serum glutamic-oxaloacetic transaminase (SGOT) 547 IU/L, SGPT 195 IU/L, alkaline phosphatase 289 IU/L and lactate dehydrogenase (LDH) 623 IU/L. Serum creatinine phosphokinase (CPK) was significantly elevated (3,469 IU/L). Serum rheumatoid factor (RF), antinuclear antibody (ANA), and anti-neutrophil cytoplasmic antibody (ANCA) were negative.

**Figure 3:**
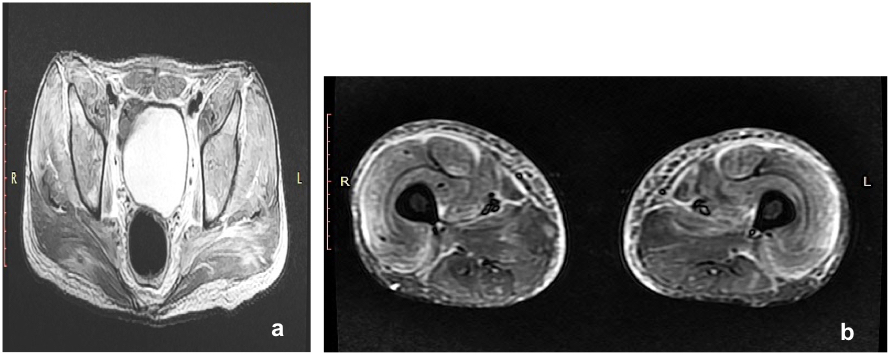
MRI of pelvis and thigh shows bilateral diffuse oedema in the muscle of the bilateral gluteal region and thigh suggestive of dermatomyositis

The chest X-ray of the patient was normal. However, magnetic resonance imaging (MRI) (Figure 3 (Fig. 3)) showed diffuse oedema in the muscle of bilateral gluteal region and thigh, appearing hyperintense on T2W and STIR images and hypointense on T1 W sequence, suggesting the possibility of dermatomyositis.

**Figure 4:**
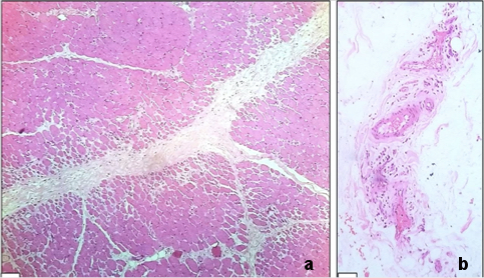
(a) Skeletal muscle fibres show perifascicular atrophy (H&E, X10); (b) areas of mild perivascular chronic inflammation are present (H&E, X10)

Electromyography and nerve conduction study results were suggestive of moderate myositis. A subsequent right thigh muscle (vastus lateralis) biopsy (Figure 4 (Fig. 4)) confirmed variation in muscle fibre size, perifascicular atrophy and perivascular chronic inflammation composed of lymphocytes and plasma cells, consistent with an inflammatory myopathy. 

Henceforth, in view of the clinical findings of the characteristic skin rash coupled with the heliotrope rash of the eyelids along with the symmetrical proximal muscle weakness, the supportive laboratory findings of raised serum CPK, SGOT, LDH levels and the pathognomic radiological and biopsy findings, a final diagnosis of juvenile dermatomyositis with bilateral Purtscher-like retinopathy (JDM with BPLR) was confirmed according to the international revised criteria of JDM [3] (Table 1 (Tab. 1)).

Subsequently, the patient was started on a high dose of intravenous methyl prednisone followed by improvement in visual acuity to 20/200 in both eyes. However, on the third day of treatment, the patient developed interstitial lung disease for which he was placed on a ventilator in the intensive care unit (ICU), where he succumbed to the illness after 2 days.

## Discussion

Juvenile dermatomyositis (JDM) is characterized by clinical features of proximal muscle weakness and skin rashes with multiple organ system involvement. Diagnosis can be made using Bohan and Peter’s [4] diagnostic criteria (a gold standard for clinical trials) or by revised criteria from 2006 based on an international consensus survey process [3]. 

Ophthalmic manifestations in JDM are infrequent with only limited citations available in the literature. The largest report of ophthalmologic complications related to JDM was a retrospective study of 82 patients [5]. Only a few published case reports have described the association of PLR with JDM, the first been reported by Bruce in 1938 [5], [6], [7], [8], [9], [10]. To the best of our knowledge, this is the first case report of juvenile dermatomyositis associated with PLR from Indian subcontinent.

PLR is characterized by Purtscher flecken, cotton-wool spots, retinal haemorrhage, and optic disc swelling. Purtscher’s retinopathy was first described in 1910 by Otmar Purtscher in a patient with severe head trauma [11]. Apart from JDM, PLR may be associated with several disorders like acute pancreatitis, preeclampsia, crush injury and fat embolism syndrome, however its association with JDM is rare. Our case is unique as the ophthalmic features preceded the dermatological and neurological features. Choi et al. have also reported an atypical presentation of the disease in which retinal changes predated any skin finding [6]. In a large review of patients with JDM, Feldman et al. concluded that heliotrope rash could be present in 66–100% of the cases [12].

Apart from the laboratory findings of raised serum enzymes, the histopathological findings in a case of dermatomyositis are peculiar and consist of varying degrees of perifascicular atrophy, vasculopathy, and perivascular inflammation. A combination of the distinctive clinical features and specific radiological, laboratory and pathological findings in JDM proves valuable in arriving at a specific diagnosis, as in our case. 

With the advancement in treatment, incidence of mortality in JDM has dramatically fallen. High-dose corticosteroids remain the mainstay of initial treatment with adjuvant therapy using immune-modulating agents such as methotrexate, intravenous immunoglobulin (IVIG), and biologic therapies including anti-tumor necrosis factor (TNF) agents, rituximab, and abatacept. However, in our case, diminution of vision with PLR presented before the onset of characteristic neurological and dermatological findings and this atypical presentation lead to a delay in the diagnosis and management of disease. As a result, there was a rapid progression of the disease resulting in the unfortunate death of the patient within a week of the diagnosis.

To conclude, primary occurrence of PLR in a case of JDM is quite rare and mandates an early diagnosis and prompt management to minimise complications. In a patient presenting with PLR, a diagnosis of JDM and other systemic manifestations should be kept in mind despite their low prevalence and atypical presentation, necessitating a meticulous examination and thorough investigation to avoid a misdiagnosis.

## Notes

### Competing interests

The authors declare that they have no competing interests.

## Figures and Tables

**Table 1:**
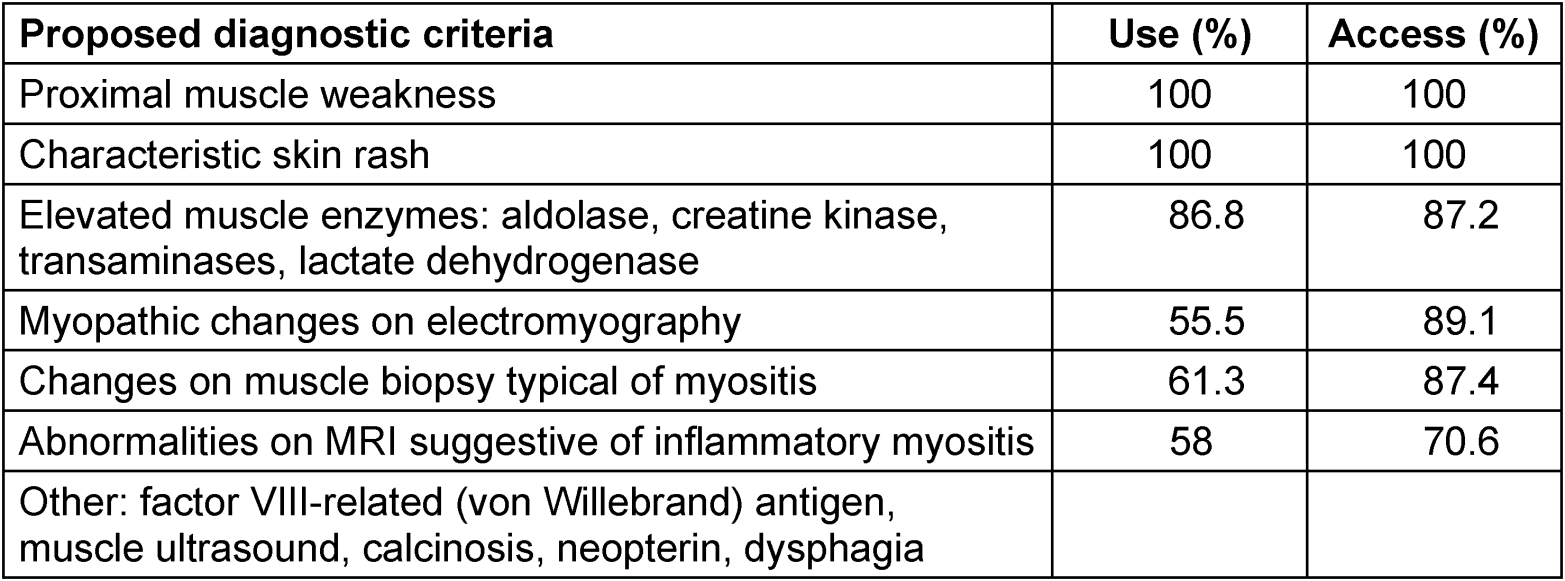
Use of and access to existing and other proposed diagnostic criteria for JDM [3]
